# Association between Municipal Health Promotion Volunteers’ Health Literacy and Their Level of Outreach Activities in Japan

**DOI:** 10.1371/journal.pone.0164612

**Published:** 2016-10-13

**Authors:** Atsuko Taguchi, Hiroshi Murayama, Sachiyo Murashima

**Affiliations:** 1 Department of Public Health Nursing, Division of Health Sciences, Tohoku University Graduate School of Medicine, Sendai, Japan; 2 Institute of Gerontology, The University of Tokyo, Tokyo, Japan; 3 Oita University of Nursing and Health Science, Oita, Japan; Kyoto University, JAPAN

## Abstract

**Objectives:**

To explore the association between health literacy and levels of three types of core activities among health promotion volunteers (developing a healthy lifestyle, outreach to family, and outreach to community members).

**Study Design:**

A cross-sectional, anonymous, self-administered postal survey of registered health promotion volunteers in the Konan area in Shiga Prefecture in Japan, conducted in January 2010. The study sample was 575 registered health promotion volunteers.

**Methods:**

The survey collected data on health literacy, gender, age, education, self-rated health, perceptions about the volunteer organization, and perceptions of recognition in the community. The level of engagement in health promotion activities was measured by the extent to which the participants engaged in seven healthy behaviors and promoted them to family members and the community. The authors compared the health literacy level and other characteristics of the participants by core health promotion activities, using a chi-squared test, to examine the associations between demographic and other variables and the three core activities (healthy lifestyle, outreach to family, and outreach to community).Logistic regression analysis was conducted to examine the association between the degree to which the volunteers engaged in core activities (“healthy lifestyle,” “outreach to family,” “outreach to community”) and the levels of health literacy (low, medium, high) among health promotion volunteers, controlling for the effects of age, gender, health condition, education which may also have an impact on volunteers’ outreach activities.

**Results:**

Four hundred and fifty-four questionnaires were returned, a 79.0% response rate. Excluding 16 cases with missing values on health literacy or the degree of health promotion activities, 438 research subjects were included in the analysis (valid response rate: 76.2%). Health literacy and a few demographic and other characteristics of the volunteers were associated with the three core health promotion activities. In bivariate analyses, active participation in the core activities was more prevalent among older volunteers (p<0.001 for all three activities). Self-rated health condition was associated with both outreach to family (p = 0.018) and community (p = 0.046). Years of experience as volunteer and perception of being recognized in the community also had statistically significant association with outreach to the community (p<0.001). In multiple logistic regression, those with higher level of health literacy were more likely than others to actively engage in outreach to family (OR = 1.70, 95% CI 1.03 to 2.80; OR = 1.76, 95%CI 1.04 to 3.00 for medium and high, respectively) and outreach to community (OR = 2.26, 95%CI 1.34 to 3.83; OR = 2.61 95%CI 1.49 to 4.58 for medium and high, respectively). Perception of being recognized in the community also had a statistically significant and positive impact on outreach to the community (OR = 1.52, 95%CI 1.17 to 1.99).

**Conclusions:**

Volunteers with higher health literacy were more likely to actively engage in outreach to family and outreach to community. Providing educational programs to improve volunteers’ health literacy may facilitate their work.

## Introduction

Japan has the most rapidly aging population in the world. As the population ages, healthcare costs will increase and so will the burden on national healthcare systems. Recently, therefore, health promotion and disease prevention in the community have become widely recognized as effective strategies to reduce healthcare costs in Japan. Implementing such strategies, however, requires efficient intervention by health professionals. Mobilizing natural helpers such as community health workers (CHWs) could be an effective way to improve health and to empower community members to improve their own health [1], [2]. CHWs are members of a community who are chosen by peers or organizations to provide basic health and medical care to their community.

According to previous studies on CHW activities, positive impact of such activities include behavioral modification, improved health status and health knowledge, access to health support among community members, and more cost-effective health and welfare services [3–5]. These outcomes were largely a result of these workers’ vigorous involvement in their community. It therefore seems important to explore strategies that could further encourage community involvement by healthcare workers.

As we have previously reported elsewhere [6], in Japan, health promotion volunteers have acted as ‘helping resources’ in the community for many years. Health promotion volunteer activity originated at the end of World War II, when a volunteer group was organized, initially to improve poor hygiene conditions. Subsequently, volunteer organizations have been established in municipalities throughout Japan to address public health problems such as high infant mortality and poor nutrition, and to prevent lifestyle-related diseases among local residents.

As community members, health promotion volunteers are expected to promote good health practices among local residents. Health promotion volunteers perform activities similar to those performed by CHWs. Each municipality recruits health promotion volunteers by flyers and word of mouth, and new volunteers are qualified after a certain period of training. How much training such volunteers receive depends on the municipality. The city where this research was conducted provides 35 hours of volunteer training which includes lectures on diet and exercise, cooking demonstration, and participation in exercise programs. Although health promotion volunteers are unpaid, their services support the community in many aspects regarding health promotion. The role of health promotion volunteers in Japan has been described fully elsewhere [6]. Despite its long history, there are few studies on volunteer health promotion activities in Japan.

More generally, in research on volunteering, antecedent and other factors related to initiating or continuing participation in volunteer activities have been studied extensively. In the volunteering process model developed by Davis et al [7], the antecedents include motivation and “dispositional empathy” toward the volunteer organization and community. Motive fulfillment and satisfaction with one’s own volunteer experiences may serve as mediating factors that will also influence whether individual volunteers continue to participate actively or not.

Among health promotion volunteers, too, volunteers’ emotional attachment to the organization or the activities does matter to whether they actively participate or not. In previous studies, the degree of anticipated satisfaction or burden associated with particular activities was related to the health promotion volunteers’ willingness to participate in those activities [8–10]. In our own previous study we have shown that volunteers’ sense of belonging to the organization is positively correlated with the level of satisfaction with their volunteer experience [11]. Another study found that health promotion volunteers were more likely to work actively when they felt that they were part of a strong community network or had a sense of belonging to the community [6].

Though today we know more about what factors are associated with active participation in volunteer activities in general and in health promotion, it is still not clear how health promotion volunteers can be encouraged to be more active and more effective in their activities. Identifying what modifiable factor drives active participation among health promotion volunteers could inform the development of educational and training program or other forms of support for such volunteers.

One significant and modifiable factor which may contribute to volunteers’ active participation in health promotion activities is health literacy. Health literacy may constitute one of the key resources for health promotion volunteers since the volunteers’ main function involves accessing and using information. Health promotion volunteers are expected to improve their own health, and promote healthy behaviors among members of their family and community. To fulfill such goals they need to have the capacity to access suitable information and to use it themselves, and also be able to communicate it to others, including family members and local residents. This corresponds with the definition of health literacy by the World Health Organization (WHO) as “the cognitive and social skills which determine the motivation to access, understand and use information in ways which promote and maintain good health” [12].

Health literacy is regarded as one of the core concepts in health promotion, and has gained increased recognition as a key to reduce disparity in access to health information [13], [14]. There are studies that demonstrate association between health care providers’ health literacy levels and patient outcomes including one that reported an improvement in health literacy among healthcare employees enhanced access to and understanding of health information by local residents [15]. To our knowledge, however, there is no empirical research examining the association between health promotion volunteers’ activities and their level of health literacy in Japan or elsewhere. The authors hypothesized that since health literacy constitutes key resource for health promotion volunteers, greater levels of health literacy would allow volunteers to more actively engage in the volunteer activities. The purpose of this study was to examine the association between health literacy and activity of health promotion volunteers.

Specifically, the main research question was:

What is the association between health literacy and levels of three types of core activity among health promotion volunteers (developing a healthy lifestyle, outreach to family, and outreach to community members)?

## Methods

### Design and Sample

A cross-sectional survey of health promotion volunteers was conducted in January 2010 in the Konan area in Shiga Prefecture in Japan. Anonymous, self-administered questionnaires were mailed directly to all registered health promotion volunteers in the Konan area, a total of 575 individuals. Completed questionnaires were mailed back to the researchers.

The Konan area includes four municipalities, and there are four health promotion volunteer associations in the area, one for each municipality. Volunteers are linked to the organization in the district or municipality in which they live, and work as a volunteer in their own communities. The Konan area is located within 15 km of Kyoto and 50 km of Osaka, and was developed as a bedroom community for those who commute to nearby major cities including Kyoto and Osaka. All four communities in this area contain long term and new residents. In January 2010, the Konan area had a population of 318 669 (160 982, 50.5% men and 157687, 49.5% women).

The survey was explained to and approved by each health promotion volunteer organization. Along with the questionnaire we sent a letter explaining the purpose of our research to each volunteer. The letter also stated that refusal to participate will have no negative implications and the survey was anonymous. The completed and returned questionnaire constituted consent. Approval for the study was obtained from the ethics committee of the Graduate School of Medicine and Faculty of Medicine at the University of Tokyo (Approval number 2870).

### Measures

#### Health literacy of health promotion volunteers

According to the WHO, health literacy is defined as “the cognitive and social skills which determine the motivation and use of information in ways which promote and maintain good health”. Nutbeam [13] proposed a model of health literacy that includes three levels:

1. functional/ basic literacy, or sufficient basic skills in reading and writing to be able to function effectively in everyday situations, broadly compatible with the narrow definition of health literacy;
2. communicative/interactive literacy, providing more advanced skills to participate actively in everyday activities, to extract information and derive meaning from different forms of communication and to apply new information to changing circumstances; and
3. critical literacy, a more advanced skill enabling critical analysis of information about life events and situations.

Among health promotion volunteers whose main role is to evaluate and communicate selected health information to community residents, we believe that the communication literacy and critical literacy to be particularly important. Being able to simply read and understand text is not sufficient. For this reason we adopted Nutbeam’s definition of health literacy in this research.

There have been other studies developing health literacy scales in Japan [16–18]. However, these largely focused on general, broadly defined health literacy, or the relationship between health literacy and self-rated health. The authors considered that this focus was too broad for our purposes which was to look at the specific health literacy required to perform effectively as a health promotion volunteer.

The authors therefore created five survey items measuring health literacy that we considered necessary for health promotion volunteers. First, functional/basic literacy was examined by a single item which asked whether the volunteers routinely “try to understand content of new information and knowledge and ask others if necessary”. Second, to assess communicative/interactive literacy, we asked the respondents if they “obtain information and knowledge from many sources” and “communicate information and knowledge to others if it seems trustworthy”. The other two items concerns critical literacy and measured the degree to which the volunteers regularly “scrutinize to what extent new information and knowledge can be trusted” and “consider how new information and knowledge can be used”. Each item was rated on a four-point Likert scale (1 = disagree; 2 = somewhat disagree; 3 = somewhat agree; 4 = agree). In all five survey items “information and knowledge” was defined as “information and knowledge necessary to participate in activities as a health promotion volunteer.” Before the survey was distributed, the authors confirmed the content validity of these items by conducting interviews with volunteers in an area other than the one in which the survey was to be done. To create the health literacy level variable, the five items were summed, and categorized into three tertiles; low, middle, and high (Cronbach’s alpha was 0.81, demonstrating a high degree of reliability).

#### Degree of engagement in core health promotion activity

A scale was developed based on Breslow’s [19]. The Breslow scale is used to assess healthy life style in Europe and the United States [20]. In Japan, Breslow’s scale with seven items is used in research [21], [22] and in screening patients at health check-ups. The degree of engagement was measured in two parts; the first part is about volunteers’ own health behaviors, the second part is about reaching out to their families and residents. First, respondents’ lifestyle was assessed, using the following seven items: no smoking, regular physical exercise, moderate or no use of alcohol, regular sleep habits, maintaining an appropriate weight, eating breakfast, and not eating between meals. The authors then asked the volunteers whether they reached out to family and local residents to promote these seven healthy behaviors. Respondents answered on a four-point Likert scale (not doing; somewhat not doing; somewhat doing; doing). Responses were dichotomized into doing (doing and somewhat doing) and not doing (not doing and somewhat not doing), and the seven responses were summed (ranging from 0 to 7). The higher the score, the healthier the lifestyle and the more vigorously they promoted healthy behaviors to family and community members.

#### Perceptions about the volunteer organization

Davis et al [7] argues that volunteers’ “dispositional empathy” contributes to more active participation in volunteer activities. Based on this model, we designed our own survey instruments to assess the volunteers’ empathy toward the volunteer organization as well as the community they belong to. Since there are no existing scales that measure such empathy among health promotion volunteers, we created one by first conducting interviews with health promotion volunteers and subsequently categorizing the responses inductively. Our scale has been improved with additional input from the research community [11]. The health promotion volunteers’ perception of how their respective volunteer organization functions was assessed by the following seven items: “The atmosphere is pleasant and we are always friendly to each other”, “There is a sense of solidarity and working together”, “The organization has an atmosphere in which it is easy to give one’s opinion”, “The organization allows anyone to ask questions freely”, “The organization has an atmosphere of mutual cooperation”, “It is easy to take days off if you need to do so for work or private reasons” and “The atmosphere is such that volunteers find it easy to participate in the activities.”. Respondents answered on a four-point Likert scale (1 = disagree; 2 = somewhat disagree; 3 = somewhat agree; 4 = agree). The scale scores ranged 7–28 with a higher score indicating more positive views of the organization. Cronbach’s alpha was 0.92, demonstrating a high degree of reliability.

#### Perception of being recognized in the community

Feelings about whether volunteers are recognized in the community were assessed by one question: “Health promotion volunteers are known to members of the community where you live.” Respondents answered on a four-point Likert scale (1 = disagree; 2 = somewhat disagree; 3 = somewhat agree; 4 = agree).

#### Health condition

The volunteers’ own health condition was self-assessed using a Likert-type scale of poor, somewhat poor, fair, and good.

#### Demographic variables

The survey asked respondents to provide data on gender, age, and educational level. Educational level was assessed by three categories: junior high school/high school, junior college/vocational school, and college/university graduate.

### Analytic Strategy

Out of the 575 questionnaires distributed, 454 were returned, a response rate of 79.0%. Excluding 16 cases with missing values on health literacy or the degree of health promotion activities, 438 research subjects were included in the analysis (valid response rate: 76.2%).

The authors compared the health literacy level and other characteristics of participants by levels of engagement in core health promotion activities, using a chi-squared test, to examine the associations between demographic and other variables with the three core activities (healthy lifestyle, outreach to family, and outreach to community). Logistic regression analysis was used to examine the association between the presence of each of the three core activities (“health lifestyle,” “outreach to family,” “outreach to community”) and the levels of health literacy (low, medium, high) among the health promotion volunteers. We examined the frequency distribution of the variables, degree of engagement in core health promotion activity, which was not normal in all three core activities. For this reason, we have decided to treat these not as continuous variables but as binary ones. To create a series of dichotomous dependent variables, the degree of engagement in a core activity (0–7) was recoded using its median value as the cutoff point. Results are presented as an odds ratio (OR) with a 95% confidence interval (95% CI). Statistical analyses were performed using two-tailed tests, and significance was set at p < 0.05. SPSS for Windows 19.0 (Japanese version) was used for statistical analysis.

## Results

Table 1 shows the characteristics of the participants in total and by the level of core health promotion activities. In total, 50.5% were 60 years old and over and 97.3% were women. In terms of education level, about two thirds, 62.8%, were high school graduates, and 27.4% were graduates from a junior college or vocational school. The distribution of level of experience was relatively even, with 37.0% having 1–4 years’ experience as a health promotion volunteer, 28.8% having 5–8 years, and 34.0% having 9 years or more. Regarding the degree of core activities, the mean and standard deviation for “healthy lifestyle” was 5.74±0.93 [range:0–7, mode:6], “outreach to family,” 5.93±1.54 [range:0–7, mode:7], and for “Outreach to community” it was 3.47±2.89 [range: 0–7, mode: 0].

**Table 1:** Characteristics of study participants by level of core health promotion activities.

			Total		Healthy lifestyle					Outreach to family					Outreach to community				
						5.74 ± 0.93					5.93 ± 1.54					3.47 ± 2.89			
					[range: 0–7, mode: 6]					[range: 0–7, mode: 7]					[range: 0–7, mode: 0]				
					Low (<6)		High (≥6)			Low (<7)		High (= 7)			Low (<3)		High (≥3)		
			n = 438		n = 151		n = 263			n = 196		n = 227			n = 189		n = 235		
			n	%	n	%	n	%	p-value	n	%	n	%	p-value	n	%	n	%	p-value
**Health literacy**																			
** **		Low	138	31.5%	47	31.1%	85	32.3%	0.562	76	38.8%	56	24.7%	0.006	84	44.4%	51	21.7%	<0.001
		Medium	158	36.1%	50	33.1%	97	36.9%		66	33.7%	88	38.8%		60	31.7%	92	39.1%	
		High	142	32.4%	54	35.8%	81	30.8%		54	27.6%	83	36.6%		45	23.8%	92	39.1%	
**Demographic variables**																			
	Age																		
		<60 years	213	48.6%	101	66.9%	106	40.3%	<0.001	119	60.7%	90	39.6%	<0.001	121	64.0%	88	37.4%	<0.001
		≥60 years	221	50.5%	50	33.1%	157	59.7%		76	38.8%	136	59.9%		67	35.4%	146	62.1%	
		Non-response	4	0.9%	0	0.0%	0	0.0%		1	0.5%	1	0.4%		1	0.5%	1	0.4%	
	Gender																		
		Male	10	2.3%	4	2.6%	6	2.3%	0.731	4	2.0%	5	2.2%	1.000	1	0.5%	9	3.8%	0.055
		Female	426	97.3%	147	97.4%	256	97.3%		192	98.0%	222	97.8%		188	99.5%	225	95.7%	
		Non-response	2	0.5%	0	0.0%	0	0.0%		0	0.0%	0	0.0%		0	0.0%	1	0.4%	
	Educational level																		
		Junior high / high school graduate	275	62.8%	88	58.3%	176	66.9%	0.238	120	61.2%	146	64.3%	0.639	109	57.4%	160	68.1%	0.050
		Junior college / vocational school graduate	120	27.4%	45	29.8%	68	25.9%		59	30.1%	59	26.0%		65	34.4%	53	22.6%	
		College graduate	33	7.5%	16	10.6%	16	6.1%		15	7.7%	17	7.5%		13	6.9%	17	7.2%	
		Non-response	10	2.3%	2	1.3%	3	1.1%		2	1.0%	5	2.2%		2	1.1%	5	2.1%	
**Self-rated health condition**																			
		Healthy	385	87.9%	133	88.1%	235	89.4%	0.659	165	84.2%	209	92.1%	0.018	160	84.7%	216	91.9%	0.046
		Not healthy	50	11.4%	18	11.9%	27	10.3%		31	15.8%	17	7.5%		28	14.8%	19	8.1%	
		Non-response	3	0.7%	0	0.0%	1	0.4%		0	0.0%	1	0.4%		1	0.5%	0	0.0%	
**Variables related to volunteer activity**																			
	Years of experience as a health promotion volunteer																		
		1–4 years	162	37.0%	68	45.0%	88	33.5%	0.064	85	43.4%	73	32.2%	0.059	90	47.6%	70	29.8%	<0.001
		5–8 years	126	28.8%	40	26.5%	82	31.2%		52	26.5%	71	31.3%		56	29.6%	66	28.1%	
		9 or more years	149	34.0%	43	28.5%	93	35.4%		59	30.1%	83	36.6%		42	22.2%	99	42.1%	
		Non-response	1	0.2%											1	0.5%	0	0.0%	
	Perceptions about the volunteer organization* [range:7–28]																		
		Low (<21)	141	32.2%	50	33.1%	82	31.2%	0.697	73	37.2%	64	28.2%	0.096	67	35.4%	69	29.4%	0.285
		High (≥21)	295	67.4%	101	66.9%	180	68.4%		123	62.8%	162	71.4%		122	64.6%	165	70.2%	
		Non-response	2	0.5%	0	0.0%	1	0.4%		0		1	0.4%		0	0.0%	1	0.4%	
	Perception of being recognized in the community*[range: 1–4]																		
		Low (<3)	230	52.5%	86	57.0%	132	50.2%	0.367	111	56.6%	113	49.8%	0.352	120	63.5%	102	43.4%	<0.001
		High (≥3)	205	46.8%	64	42.4%	130	49.4%		84	42.9%	112	49.3%		68	36.0%	131	55.7%	
		Non-response	3	0.7%	1	0.7%	1	0.4%		1	0.5%	2	0.9%		1	0.5%	2	0.9%	

Results of Chi-squared test.

*Higher value indicates more favorable perception.

Health literacy and a few demographic and other characteristics of the volunteers were associated with the three core health promotion activities. Those with higher level of health literacy were more likely than others to actively engage in outreach to family (p = 0.006) and outreach to community (<0.001). Active participation in the core activities was more prevalent among older volunteers (p<0.001 for all three activities). Selfrated health condition was associated with outreach to family (p = 0.018) and community (p = 0.046). Years of experience as volunteer and perception of being recognized in the community also had statistically significant association with outreach to the community (p<0.001).

Table 2 shows the relationship between the degree of activity (“healthy lifestyle”, “outreach to family”, “outreach to community”) and the level of health literacy. Logistic regression analysis showed that “outreach to family” was associated with medium (odds ratio (OR) = 1.70, 95% CI: 1.03–2.80) and high health literacy (OR = 1.76, 95% CI: 1.04–3.00), as was “outreach to community” (OR = 2.26, 95% CI: 1.34–3.83 for medium and OR = 2.61, 95% CI: 1.49–4.58 for high). Increased “outreach to community” was also associated with nine or more years of experience (OR = 1.87, 95% CI: 1.09–3.21) and the feeling that volunteers were recognized in the community (OR = 1.52, 95% CI: 1.17–1.99). There was no association between health literacy and one’s own healthy lifestyle.

**Table 2:** Results of logistic regression analysis for core health promotion activities.

			Healthy lifestyle(n = 405)			Outreach to family(n = 405)			Outreach to community(n = 411)		
			β	OR	95% CI	β	OR	95% CI	β	OR	95% CI
**Health literacy (ref. = Low)**											
	Medium		-0.10	0.91	(0.53‐1.55)	0.53	1.70	(1.03‐2.80)	0.82	2.26	(1.34‐3.83)
	High		-0.53	0.59	(0.33‐1.03)	0.57	1.76	(1.04‐3.00)	0.96	2.61	(1.49‐4.58)
**Demographic variables**											
	Age (ref. = ≤60)		1.14	3.14	(1.96‐5.02)	0.69	1.99	(1.29‐3.06)	0.69	2.00	(1.27‐3.13)
	Gender (ref. = female)		-0.41	0.66	(0.17‐2.65)	-0.21	0.81	(0.20‐3.28)	1.68	5.35	(0.63‐45.03)
	Education level (ref. = Junior high school / high school graduate)										
** **		Junior college / vocational school graduate	-0.08	0.92	(0.57‐1.50)	-0.07	0.93	(0.59‐1.48)	-0.50	0.61	(0.37‐0.99)
		College graduate	-0.50	0.60	(0.27‐1.35)	-0.10	0.91	(0.41‐2.02)	-0.21	0.81	(0.34‐1.95)
**Self-rated health condition**											
		Healthy (ref. = not healthy)	0.29	1.34	(0.98‐1.83)	0.26	1.30	(0.97‐1.75)	0.27	1.31	(0.95‐1.79)
**Variables related to volunteer activity**											
	Years of experience as a volunteer (ref. = 1–4 years)										
		5–8 years	0.24	1.28	(0.75‐2.17)	0.23	1.26	(0.76‐2.09)	0.15	1.16	(0.69‐1.98)
** **		9 or more years	0.13	1.14	(0.66‐1.96)	0.08	1.09	(0.65‐1.81)	0.63	1.87	(1.09‐3.21)
	Perceptions about the volunteer organization* [range: 7–28]		0.00	1.00	(0.94‐1.05)	0.02	1.02	(0.97‐1.08)	0.00	1.00	(0.95‐1.06)
	Perception of being recognized in the community* [range: 1–4]		0.06	1.06	(0.81‐1.38)	0.09	1.10	(0.86‐1.41)	0.42	1.52	(1.17‐1.99)
									

β: Regression Coefficient

OR: Odds Ratio

CI: Confidence Interval

*Higher value indicates more favorable perception.

## Discussion

The results showed that volunteers with higher health literacy were significantly more active in outreach to family and the community. This suggests that such outreach activity could be enhanced by improvement in health literacy among the volunteers. Health promotion volunteers need to be able not only to access information and make use of it themselves, but also to communicate it to others, such as family members or local residents. High health literacy may allow health promotion volunteers to fulfill their role more actively. The authors suggest that high health literacy may give volunteers more confidence to reach out to others, and also by reaching out volunteers may be able to use their skills and thereby build greater confidence.

The authors expected to find a correlation between the level of health literacy and living a healthy lifestyle among the volunteers, but we did not find any association between the two. Our results appear to suggest that volunteers are more likely to communicate new and relevant information to family and community members than to draw on it for themselves. It is possible, however, that compared with the general population, health promotion volunteers already have a greater interest in health and have cultivated healthy habits. That is, only a basic knowledge of health literacy is necessary to live a broadly healthy lifestyle, as measured by the seven aspects considered in this study. Subjects in this study had an average health literacy score of 5.74 ± 0.93 out of 7(Mode: 6), which is quite high compared to the results of a few studies conducted in Japan which report the modal value of five. [21], [22]. It may be that health promotion volunteers’ prior knowledge, which they used to develop healthy habits, links to health information provided through their voluntary activities. This might underestimate the association between health literacy and personal efforts to develop a healthy lifestyle. However, developing a healthy lifestyle among health promotion volunteers is also one of the aims of health promotion activity, and it may also allow volunteers to serve for a long time. Efforts to provide appropriate information that will challenge health promotion volunteers to further improve their own behavior may be helpful.

The analysis also considered factors other than health literacy that could encourage health promotion activity. Years of experience as a volunteer was associated with increased outreach to members of the community. At present, voluntary organizations face a problem, in that quite a number of volunteers resign after only a short time [23], [24]. Our finding that greater experience tends to lead to greater outreach to family and community reinforces the benefits of long-term volunteering to the organization. It is therefore necessary to ensure that organizations support volunteers who have work or family commitments so that they can continue to volunteer.

Volunteers who felt acknowledged by the community tended to reach out more vigorously to community members. This result is consistent with previous studies [10]. It is, however, difficult for voluntary organizations alone to win recognition from the community [23–25]. Public bodies have a classic function to spread information among local residents. If local government and similar organizations helped health promotion volunteers by publicizing their activity, and therefore improving recognition, this could lead to a more effective and collaborative relationship.

### Limitations

This study has several limitations. First, the scale used to measure health literacy was developed for this study, and its reliability and validity have not been externally examined. There are scales developed to measure health literacy among the Japanese population [16–18], but they are too general for the purpose of this study. Further investigation is required, either to verify and measure the health literacy of health promotion volunteers using a scale with greater reliability and validity, or to assess the reliability and validity of the scale used in this study. Second, the study was cross-sectional, so we cannot definitively say if the observed association between the volunteers’ health literacy and their activity levels is causal or not, and if there was a causal arrow whether it is in an expected direction or not. In the future, to show that higher levels of health literacy lead to greater levels of outreach activities with greater confidence, we need multiple waves of observations to observe how changes in the level of health literacy affect health promotion activities.

Third, this study was conducted only in Japan, and was limited to one area. To increase the generalizability of the findings, it is necessary to carry out similar studies in different settings.

Fourth, non-response bias cannot be ruled out. Those who declined to participate may not be active volunteers. Additionally, there may be recall bias in the responses to health behavior questions.

### Future research and practical implications

Our results suggest that improving volunteers’ health literacy would facilitate their outreach towards family and community members. The authors therefore believe that it is recommended to provide assistance or educational programs to improve the health literacy of health promotion volunteers. A study conducted in Israel describes an approach to improve health professionals’ communication skills by using face-to-face literacy [26], [27]. An interactive education program to improve communication skills was offered to doctors, nurses, and nutritionists on site, which improved both quantitative and qualitative knowledge, skill, and satisfaction. These studies highlight the need to improve service providers’ communication skills.

Both initial and on-the-job health promotion volunteer training in Japan are focused on providing knowledge about disease prevention and improving health. How best to communicate these concepts in an easy-to-understand manner to family and community members are left to the individual volunteer. The authors suggest that a training program on how to communicate health information in an approachable and effective way would increase the efficacy of health promotion volunteers’ activity. For example, increasing the number of hours allocated to role playing and simulation in health education during the on the job training (OJT) may be one such strategy.

Future research should focus on developing and validating an effective health literacy training program for health promotion volunteers. Furthermore, the vast majority of the health promotion volunteers who participated in the study are female. In general, women tend to be overrepresented among the health promotion volunteers trained by Japanese municipalities. In the future, male volunteers’ behavior and orientations must also be examined to generalize our findings.

## Supporting Information

S1 File SAV file. The data set for the analysis.
(SAV)
